# Evaluation of Antimicrobial and Antifungal efficacy of Chitosan as endodontic irrigant against *Enterococcus Faecalis* and *Candida Albicans* Biofilm formed on tooth substrate

**DOI:** 10.4317/jced.53210

**Published:** 2017-03-01

**Authors:** Pankaj Yadav, Sarika Chaudhary, Rajendra K. Saxena, Sangeeta Talwar, Sudha Yadav

**Affiliations:** 1 Senior Resident Associate Professor, Department of Conservative Dentistry and Endodontics, Maulana Azad Institute of Dental Sciences, New Delhi, India; 2 Associate Professor, Department of Conservative Dentistry and Endodontics, Maulana Azad Institute of Dental Sciences, New Delhi, India; 3 Professor Department of Microbiology, University of Delhi South Campus; 4 Professor and HOD Department of Conservative Dentistry and Endodontics, Maulana Azad Institute of Dental Sciences, New Delhi, India; 5 MDS, Senior resident, Department of conservative dentistry and endodontics, Maulana Azad Institute of Dental Sciences, New Delhi, India

## Abstract

**Background:**

Bacterial biofilms formed on the root canal wall are often difficult to remove. This study aimed to evaluate the cytotoxic effect and antibacterial efficacy of chitosan when used as root canal irrigant against *E. Faecalis* and *Candida albicans* biofilm formed on tooth substrate.

**Material and Methods:**

The present study evaluated antibacterial effect of 0.25% Chitosan, 0.5% Chitosan, 2% chlorhexidine and 3% sodium hypochlorite against *Enterococcus faecalis* and *Candida Albicans*. Agar-well diffusion methods, minimal inhibitory concentration tests and biofilm susceptibility assays were used to determine antibacterial activity. Teeth specimens were sectioned to obtain a standardized tooth length of 12mm. Specimens were inoculated with 10 mL of the freshly prepared *E. Faecalis* suspension and *Candida albicans* for 4 weeks. The specimens were then instrumented with ProTaper rotary files F3 size. After irrigation with test solution, three sterile paper points were placed into one canal, left for 60 s and transferred to a test tube containing 1 mL of reduced transport fluid. The number of CFU in 1 mL was determined.

**Results:**

3-week biofilm qualitative assay showed complete inhibition of bacterial growth with 3% Sodium hypochlorite, 2% Chlorhexidine and Chitosan except saline, which showed presence of bacterial growth. Significant reduction of colony forming units (CFU)/mL was observed for the chitosan groups and the antibacterial activity of the chitosan groups was at par with 3% NaOCl and 2% Chlorhexidine. It was observed that the chitosan showed no cytotoxicity at 3mg/ml and 10% cytotoxicity at 6mg/ml.

**Conclusions:**

The use of chitosan as a root canal irrigant might be an alternative considering the various undesirable properties of NaOCl and chlorhexidine.

** Key words:**Biofilm, Candida albicans, Chitosan, Cytotoxicity, Enterococcus faecalis.

## Introduction

The persistence and growth of bacteria in root canal system is the main causative factor in pulpal and periradicular lesions (1). The success of the endodontic treatment primarily depends on successful removal of microbes from the infected root canal system (2). Although bacteria are the main microorganisms found in primary endodontic infections, there are some studies which state the presence of fungi in infected root canals (3). *Candida albicans* and *Enterococcus faecalis* are considered the most resistant species of fungi and bacteria which are responsible for root canal treatment failures (4). The collagenolytic activity of *C. albicans* promotes colonisation in the root canal as it uses dentin as a nutrient source leading to its high virulence (5).

Chemomechanical preparation plays a major role in disinfection by causing a drastic reduction in the bacterial populations located in the main root canal (6). The choice of an irrigant depends on their effectiveness to act as lubricants during instrumentation, flush debris and smear layer and efficacy on virulent bacteria present in the canal. Variations in chemical formulations of various irrigants might also have different impact with pulp, necrotic tissues and microorganisms (7). Sodium hypochlorite (NaOCl) is the most widely used irrigant due to its antimicrobial and organic tissue dissolving ability but it is toxic to the periapical tissues and weakens dentine by reducing its flexural strength and resilience by making it more susceptible to deformation and possibly fractures (4).

Chlorhexidine has been suggested as efficient alternative to NaOCl. Chlorhexidine gluconate has been widely used in dentistry as an antimicrobial rinse in periodontics and as an effective irrigant in endodontics (8). Chlorhexidine shows broadspectrum antibacterial effect, extended residual activity and a relative absence of toxicity (9). The use of CHX as an endodontic irrigant is restricted because it can discolor teeth and also lacks tissue dissolving ability. Other side effects include loss of taste, burning sensation of the oral mucosa, subjective dryness of the oral cavity and discoloration of the tongue (8).

The search for new alternatives is necessary considering the disadvantages of the available antimicrobial irrigants. Chitosan is a natural polysaccharide which is biocompatible, biodegradable, shows bioadhesion and lacks toxicity (9). Chitosan is a cationic biopolymer that possesses lasting antibacterial properties and low production costs (10). Chitosan is obtained by the deacetylation of chitin, which is found in crab and shrimp shells (11).

To date, none of the study has consistently investigated the effects of chitosan against these microorganisms. So the aim of the study was to evaluate the antibacterial and cytotoxic effect of Chitosan a new alternative when used as root canal irrigant and to compare its action to the commonly used root canal irrigants sodium hypochlorite and chlorhexidine.

## Material and Methods

*Candida albicans* was cultivated in Sabouraud Dextrose broth and *E. faecalis* (ATCC 29212) culture was prepared in Mueller-Hinton Broth (MHB) and the turbidity was adjusted to 0.5 McFarland standard to obtain a cell density of 1.5×108 cells/mL. Antimicrobial activity of extracts against *E. faecalis* and *C. albicans* was evaluated using agar diffusion, microdilution and biofilm susceptibility tests.

-Test solutions preparation

For preparation of the 0.25% and 0.5% chitosan (Mahtani Chitosan Pvt. Ltd Veraval, India) solution, 0.25 g and 0.5g of chitosan was diluted in 100 mL of 1% acetic acid and the mixture was stirred for 2 h using a magnetic stirrer until obtaining crystalline homogeneous solutions with 3.2 pH.

-Antibacterial Sensitivity 

Well diffusion method was used for the qualitative assay of Antibacterial Activity of the samples. The bacterial strain *E. faecalis* MTTCC 9221 and Yeast strain *Candida albicans* were used in the present study. The broth cultures were swabbed on sterile Mueller-Hinton agar plates using sterile swabs. The wells per plate were cut into agar plates with the help of sterile cork-borer and 100 µl of test samples (0.25% Chitosan, 0.5% chitosan, 2% Chlorhexidine, 3% Sodium Hypochlorite and Saline) were added to the wells respectively. The plates were incubated at 37°C for 24h. The antimicrobial activity was evaluated by measuring the diameter of inhibition zone. All procedures were carried out in triplicate in a laminar flow chamber under aseptic conditions.

-IC50 and MIC determination

**Figure 1:**
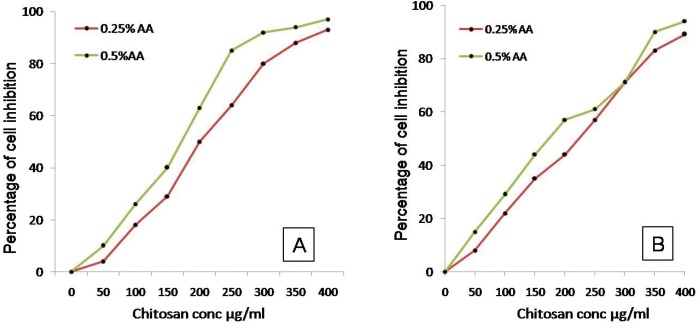
A) and B) show the percentage of cell inhibition by chitosan for *E. faecalis* and *C. albicans*.

The inhibitory concentration 50% and minimum inhibitory concentration (MIC) of the Chitosan were determined by the tube dilution method. Double dilution was made from a higher dilution 5 mg/mL to a lower dilution in a series of test tubes. Each tube was inoculated with bacterial suspensions and incubated at 37ºC for eight hours. The MIC was regarded as the lowest concentration in the series of dilutions, which did not permit the growth of the susceptible bacteria. The antibacterial activity of Chitosan was determined by the CFU assay. Briefly, the two potential pathogenic cultures, *E. faecalis* MTTCC 9221 and *Candida albicans* (evaluated on the basis of agar diffusion method) were grown to mid-log phase (A600, 0.8) (Fig. 1). 5 ml of bacterial culture were incubated at 37ºC with different concentrations of Chitosan (0.5 to 5 mg/ml), and aliquots of the assay mixture were incubated for 4 hours. Then the chitosan samples inoculated with test cultures were serially diluted with 10 mM phosphate buffer saline (pH 7.4) and 150 μl of each was plated on nutrient agar plates and incubated at 37ºC overnight. 2 % Chlorhexidine and 3% Sodium Hypochlorite was used as +ve control and double distilled water (DDW) was used as negative control. The test solutions were tested in duplicate utilizing the first bacterial magnification.

-Cytotoxicity assay

Centrifugation of fresh blood was done, plasma abstracted and packed cell volume of red blood corpuscles was obtained. Red blood corpuscles were washed with saline and centrifuged an abundance of times to abstract white cells and any traces of plasma. 1 ml of packed cell was added to 4 ml of saline to increase the volume of blood to 5 ml. A total volume of 100µl of this diluted red blood corpuscles was added to 18 test tubes with six test tubes in each group.

For all the three (Chitosan, Chlorhexidine and hypochlorite) groups, the first test tube was kept as a control in which no irrigant was added. In the second test tube 10 μl of the irrigant was integrated. 20 μl was integrated to the third test tube, 30 μl to the fourth test tube, 40 μl to the fifth test tube and 50 μl to the test tube. After an incubation time of 3 min, hemoglobin percentage after hemolysis of red blood corpuscles was noted utilizing an automated hemoanalyzer (ABX Micros 60, HORIBA ABX, Japan). The hemoanalyzer quantified the intracellular hemoglobin content of the remaining red blood corpuscles after hemolysis. For all the three groups, the experiment was reiterated 3 times and the mean value was taken.

-Grouping and assessment protocol

The samples were randomly divided into five experimental groups with 10 samples each for *E. faecalis* and Candida and irrigated with 1 ml of each irrigant for 10 min.

GroupA: 0.25% Chitosan (pH 3.2)

Group B: 0.5% Chitosan (pH 3.2) 

Group C: 2% Chlorhexidine 

Group D: 3% Sodium Hypochlorite

Group E: Saline 

-Biofilm formation on the root canal

Single-rooted human mandibular premolars with fully formed apices were used in this study. The teeth were cleaned of superficial debris, calculus and tissue tags and stored in normal saline to avert dehydration afore use. Teeth were radiographed to corroborate the presence of a single patent canal. The tooth specimens were sectioned below the cementoenamel junction with a diamond disc to obtain a standardized tooth length of 12mm for uniform specimen. The roots were rinsed with distilled water and sterilized in an autoclave at 121°C for 20 min. After sterilization, the specimens were incubated in BHI for 24 h at 37°C to confirm that there was no bacterial contamination. Specimens were inoculated with 10 mL of the freshly prepared *E. faecalis* and *Candida albicans* suspension and incubated aerobically in a glass test tube at 37°C for 4 weeks to allow biofilm formation. The medium was refreshed every 3 days. Random sampling and gram staining confirmed the viability and purity of the *E. faecalis* culture. After four weeks, five specimens were randomly selected and visualized by scanning electron microscopy (SEM) to confirm the formation of the *E. faecalis* biofilms.

-Bacterial quantification

Specimens from *E. faecalis* and *Candida albicans* group were treated according to the type of irrigant used during instrumentation. All procedures were performed under aseptic conditions. The root canals were negotiated and instrumented to a size 10 K file (Dentsply Maillefer) without irrigation. The specimens were instrumented with ProTaper rotary files (Dentsply Maillefer) using a crown-down technique, and the canals were enlarged to an F3 ProTaper rotary file using the sequence recommended by the manufacturer. During instrumentation, the canals were irrigated with 1 mL of the relevant irrigating solution for 10 min between each instrument change. The solutions were introduced into the canals using a 27-gauge needle. After irrigation, three sterile paper points were placed into one canal, left for 60 s and transferred to a test tube containing 1 mL of reduced transport fluid.

Samples were vortexed for 10 s and 10-fold diluted. Aliquots (0.1 mL) of samples were plated onto BHI agar plates and incubated at 37°C for 48 h. The number of CFU in 1 mL was determined. *E. faecalis* colony counts were transformed to log10 values to normalize the data.

-SEM observations

**Figure 2:**
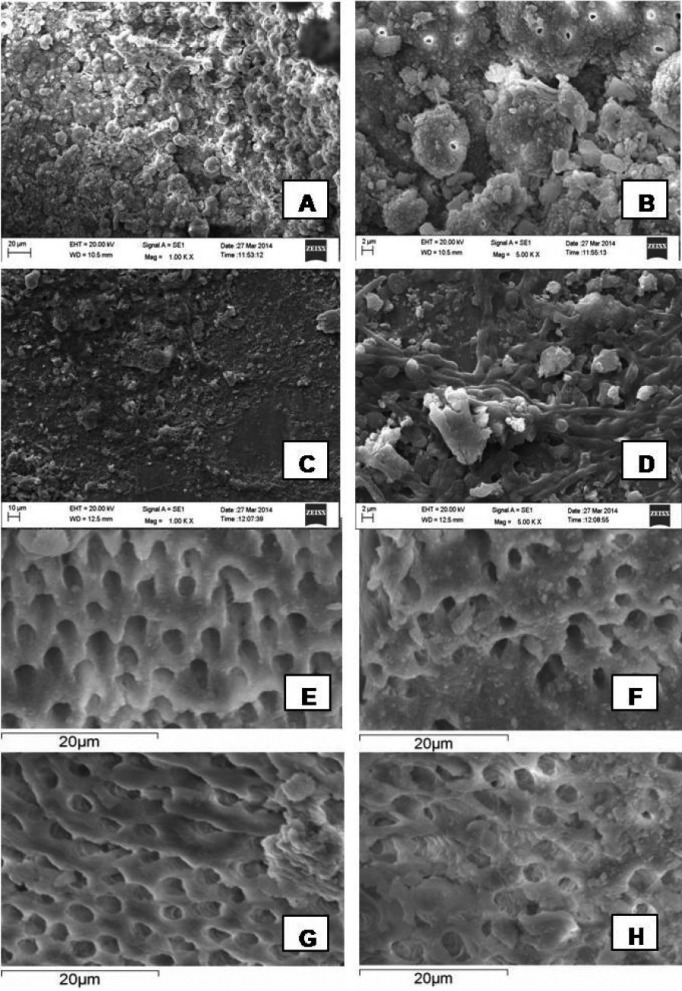
Photomicrograph of root canals contaminated with *E. faecalis* and *C. albicans*. A) *E. faecalis* 1000X ; B) *E. faecalis* 5000X; C) *C. albicans* 1000X; D) *C. albicans* 5000X. E-H) shows SEM images of the inhibition of *E. faecalis* biofilm formation (residual activity) with final irrigation regimens on teeth samples: E) 3% NaOCl,; F) 2% Chlorhexidine; G) 0.5% Chitosan and H) 0.25% Chitosan.

The specimens were dried, mounted on metallic stubs, gold sputtered and evaluated by SEM ((JSM5410; JEOL, Tokyo, Japan) micrographs with X1000 and X5000 magnifications (Fig. 2A-D). Two diametrically opposed grooves were made in the teeth utilizing metallic discs under cooling and a bi-bevel chisel was habituated to split the teeth in half lengthwise. The hemisected side with fewer irregularities, which best represented the total root canal length, was selected. These areas were utilized for the SEM analysis by the specialist.

-Statistical analysis

Statistical analysis was performed by using SPSS version 11.5. Along with descriptive analysis, one-way analysis of variance (ANOVA) with Tukey’s post hoc testing was done to evaluate the overall significance of CFU/mL between and within the different test groups for both *E. Fecalis* and *C. albicans*. *P* < 0.05 (95% confidence level) was considered statistically significant.

## Results

-Agar diffusion test 

The test irrigants showed large zones of inhibition against *E. faecalis* and *Candida albicans* in the agar diffusion test. Saline showed no antibacterial activity. Inhibitory effects were dependent on concentrations and ranked from the strongest to the weakest solutions as follows for *E. faecalis*: 2% Chlorhexidine, 3% NaOCl and Chitosan. The means of the diameters of the zones of inhibition are shown in  Table 1. The size of the zone of inhibition between NaOCl and CHX was not statistically different (*p* >0.05) ( Table 1).

**Table 1:**
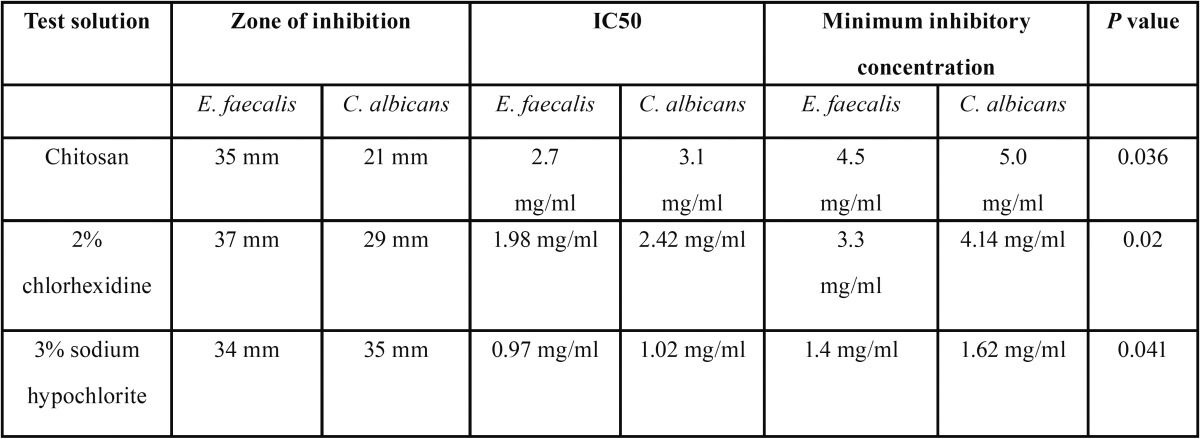
Antimicrobial activity and zone of inhibition.

-Microdilution test 

The minimum inhibitory concentration of chitosan on *E. faecalis* growth in test tubes was found to be 4.5mg/ml. Thereafter, a 0.5% and 0.25% solution of Chitosan diluted in 1% acetic acid was used in comparison with the other treatments.

-Biofilm susceptibility assay

**Figure 3:**
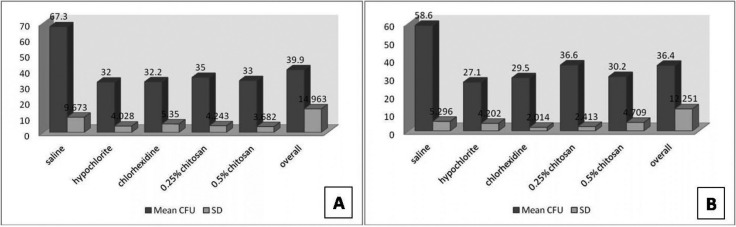
A) and B) shows Antibacterial activity of Chitosan, Chlorhexidine and Hypochlorite against *E. faecalis* and *C. albicans* respectively.

The saline group had the highest mean bacterial count of 67.30 CFU/ml for *E. faecalis* (Fig. 3A). For each of the test medications there was a significant decrease in bacteria compared with the saline group. For *C. albicans* 3% sodium hypochlorite produced the lowest bacterial counts of any of the test medications (Fig. 3B). The antimicrobial effects of the test irrigants were ranked from strongest to weakest as follows: 3% NaOCl, 2% CHX and 0.5% Chitosan followed by 0.25% Chitosan and saline.

-Cytotoxicity assay

It was observed that the chitosan showed no cytotoxicity at 3mg/ml and 10% cytotoxicity at 6mg/ml. The 2% chlorhexidine showed no cytotoxicity at 10% conc, 3% sodium hypochlorite was 72% cytotoxic at 10% conc.

## Discussion

*E. faecalis* is a facultative microbe that is nonfastidious, easy-to-grow, ability to form monospecies biofilms and progressively and rapidly colonizes tubules. *E. faecalis* is about nine times more likely to be present in teeth with primary infections that has been root canal treated (12). *E. faecalis* possess different virulence factors that avail their adhesion to host cells and extracellular matrix, which in turn facilitates tissue incursion, causes immunomodulation and engenders toxin mediated damage (14). Various virulence factors associated with *E.faecalis* may compete with other microorganisms and resist nutritional deprivation in treated canals (14). It exhibits strong adhesion to collagen (15) and display resistance to chemomechanical preparation (16). It can also survive in a quiescent phase with low metabolic activity for a long period of time (17). Three weeks old *E. faecalis* biofilm on dentinal canal was preferred in the present study to replicate their usual endodontic significance (18). Longer incubation times may result in a biofilm which is difficult to remove and may influence its susceptibility to disintegration (19).

The root canals of teeth with pulp necrosis, especially those with persistent endodontic infections harbour most commonly the *C. albicans* fungal species. *C. albicans* forms biofilm as it also has ability to colonize dentinal walls and penetrate into tubules (20).

The choice of culture media in the present study was Sabouraud Dextrose broth and Mueller-Hinton Broth, as these media are readily available and commonly used for *C. albicans* and *E. faecalis*. In our study to determine the antimicrobial efficacy of the test irrigants agar diffusion test was used. Agar diffusion test is an accepted and standardized method making it reproducible, simple to perform and relatively inexpensive (21).

The utilization of chitosan is justified as alternative option of an irrigating agent with antimicrobial potency. Chitosan’s antibacterial nature is due to the interaction between positively charged chitosan and a negatively charged bacterial cell which transmutes the bacterial cell permeability, leading to the leakage of intercellular components and cell death (22). Chitosan binds to DNA and inhibits mRNA synthesis by penetrating toward the nuclei of microorganisms and interfering with the synthesis of mRNA and proteins (22).

Sodium hypochlorite has high surface tension averts direct contact of the irrigant with the dentinal walls of the anatomical complexities (23). The use of sodium hypochlorite has risk of extrusion into periapical tissues causing inflammation, ecchymoses, hematoma and sometimes even necrosis and paresthesia (24). Chlorhexidine is an efficacious endodontic irrigant due to its anti-microbial activity against Gram-positive and Gram-negative organisms (4).

3%NaOCl, 2%CHX and .25% & .5% chitosan were the antimicrobial agents selected in this study to test their efficacy. Numerous studies have evaluated the antimicrobial effects of NaOCl and CHX in endodontic treatment (4,25,26). NaOCl and chitosan successfully removed and disintegrated the biofilm formed on the surface of the root canal (Figs. 2E,G,H) whereas CHX was found to be less effective on biofilm (Fig. 2F). The present study used SEM as an analyzing tool to assess the formation and the response to the various antimicrobial substances on biofilm. The saline group was the least effective irrigating solution.

The results clearly demonstrated that the action of test irrigants could reduce the number of bacterial cells from the root canal. Bacterial reduction was significantly superior when NaOCl was used as irrigant. In addition to the mechanical effects, NaOCl possesses chemical effects that help in the elimination of bacteria from the root canals. The present study demonstrates the anti-bacterial efficacy of chitosan almost equivalent to 3% NaOCl, which may well be replaced by this potential animal extract as endodontic irrigant to overcome the deleterious effects of the conventional irrigants (NaOCl and chlorhexidine) on dentine. Na-OCl is known to be cytotoxic to tissues and a need for replacement with a more biocompatible irrigant is necessitated.

## Conclusions

Under the limitations of this study, it was concluded that:

1. The antibacterial activity of the chitosan groups was at par with 3% NaOCl and 2% Chlorhexidine.

2. It was observed that the chitosan showed no cytotoxicity at 3mg/ml. The 2% chlorhexidine showed no cytotoxicity at 10% conc, 3% sodium hypochlorite was 72% cytotoxic at 10% conc.

3. The use of chitosan as a root canal irrigant might be an alternative considering the various undesirable properties of NaOCl and chlorhexidine.
